# Government subsidy research on the application of IoT technology in contract-farming supply chain

**DOI:** 10.1371/journal.pone.0327816

**Published:** 2025-07-31

**Authors:** Xideng Zhou, Yuan Peng, Bing Xu, Wan-zhi Xiong, Xiao-mei Peng

**Affiliations:** 1 School of Economics and Management, Yuzhang Normal University, Nanchang, China; 2 School of Computer Science and Engineering, Jiangxi Agricultural University, Nanchang, China; 3 School of Public Policy and Administration, Nanchang University, Nanchang, China; 4 Nanchang Shenzhen Agricultural Products Center Wholesale Market Co., Ltd, Nanchang, China; Zhengzhou University of Light Industry, CHINA

## Abstract

Existing research have not yet focused on the mechanism of Internet of Things(IoT) technology’s impact on the contract-farming supply chain.We introduce the IoT technology into the contract-farming supply chain with financing, successively construct decision models for the contract-farming supply chain under the scenarios of no subsidy policy, interest rate subsidy policy, and cost subsidy policy. The results show that an increase in bank interest rate will reduce the level of IoT application, purchasing price, members benefits. When the financing interest rate is within a certain range, members benefits in the case of interest rate subsidy policy are greater than the corresponding values in the case of cost subsidy policy. In the case of no subsidy policy, interest rate subsidy policy, and cost subsidy policy, the application level of the IoT, purchasing price, and the company benefit are positively correlated with the output effect factor of the IoT technology. The farmer will actively invest in the IoT only when the cost-reducing effect of IoT is substantial. When the government’s subsidy expenditure is equal, if the goal is to increase social welfare, the government should adopt the interest rate subsidy policy; if the goal is to promote the popularization of IoT and help impoverished farmers, the government should adopt the cost subsidy policy.

## 1. Introduction

The market-driven development of contract farming is a crucial strategy for resolving the supply-demand imbalance of agricultural products, optimizing the agricultural industry structure, achieving agricultural industrialization, and accelerating the high-quality development of agriculture. Contract farming, a business model where a company or intermediary organization enters into a legally binding contract with farmers for the purchase and sale of agricultural products prior to production, clearly defines the rights and obligations of both parties. Farmers organize production in accordance with these contracts, and companies purchase agricultural products based on them. Contract farming not only enhances farmers’ productivity but also secures the raw material supply for downstream enterprises, earning high praise from agricultural production and operation entities. Due to variations in organizational models and structures, contract farming has become increasingly diverse with the “company+farmer” model being the most prevalent.

Technology is the primary productive force, and the modernization of agricultural technology is a vital foundation for agricultural modernization. As a leading mode of agricultural industrialization in China, the application of innovative technologies in contract agriculture plays a significant role in promoting the modernization of contract agriculture. Agricultural technology, relying on technological innovation, effectively addresses issues such as high agricultural costs and low production efficiency [1], providing technical support for high-quality agricultural development [2]. Among these technologies, agricultural IoT (Internet of Things) technology is instrumental in facilitating the transition of agriculture from extensive growth to technology-driven growth [3]. IoT technology can collect real-time data on agricultural production environmental parameters and the growth status of crops and livestock through various sensing devices, using this data as variables for automatic control in agricultural facilities and equipment. It has improved production efficiency and reduced the ecological damage caused by excessive use of fertilizers and pesticides [4]. In particular, the application and promotion of IoT-enabled smart irrigation technology have achieved efficiency, water conservation, land-saving, fertilizer-saving, environmental protection, and ease of management. These benefits have effectively supported the development of high-efficiency agriculture in China [5]. For example, Shandong Qihan Biotechnology Co., Ltd., has comprehensively optimized the planting, production, and sales processes of shiitake mushrooms through digital means and has achieved real-time monitoring and control of the greenhouse environment using Internet of Things (IoT) technology [6].However, the high investment cost of IoT technology hinders its widespread adoption and application, dampening farmers’ enthusiasm for modern production methods.

Agricultural subsidies are an important part of government subsidies and play a significant role in increasing farmers’ enthusiasm for grain production, raising farmers’ income, and promoting agricultural modernization. To foster the development of the agricultural industry, governments typically introduce a variety of subsidy policies [7]. For instance, since 2004, China has successively implemented the three subsidies for agriculture (direct grain subsidy, comprehensive agricultural material subsidy, and improved variety subsidy) and the minimum purchase price policy for grain, which have had positive effects [8]. Agricultural machinery purchase subsidies are an important part of the national three subsidy policies aimed at strengthening agriculture and benefiting farmers. Interest rate subsidies are government subsidies for agricultural loan interest rates. For example, in 2020, the Hunan Provincial Finance supported the Provincial Agricultural Credit Financing Guarantee Company and relevant banks in conducting policy-based agricultural machinery purchase guarantee loan business. Qualified modern agricultural machinery cooperatives, large agricultural machinery owners, and other agricultural business entities were granted a 50% loan discount based on the market-quoted interest rate and a 1% annualized guarantee fee subsidy to purchase agricultural machinery.

(**Research Gap**) Existing studies have explored the positive impact of IoT technology on agriculture from a qualitative perspective. However, there is a lack of in-depth quantitative analysis of the specific mechanisms by which IoT technology affects the order-based agricultural supply chain. Particularly, there is limited research on the appropriate government subsidy models when farmers finance IoT technology.(**Research Objectives**) This paper aims to integrate IoT technology into the order-based agricultural supply chain, which consists of a single farmer and a company, and incorporate a bank financing model. We construct decision-making models for three scenarios: no subsidy, interest rate subsidy, and cost subsidy, from a quantitative perspective. By examining the decision-making behaviors and performance changes of supply chain members under different subsidy models, we analyze the impact of government subsidy policies on the application level of IoT technology, the purchase price of agricultural products, and the profits of farmers and companies. We also explore the optimal government subsidy schemes under different policy objectives. (**Research Significance**)The significance of this study lies in constructing decision-making models to quantify the impact of different subsidy models on the decision-making and performance of supply chain members. This provides a scientific basis for the government to formulate precise subsidy policies, helps improve the efficiency of subsidy fund utilization, promotes the widespread application of IoT technology in order-based agriculture, enhances the profits of farmers and the overall performance of the agricultural supply chain, and contributes to the modernization of agriculture.

## 2. Literature review

This article takes the order-based agricultural supply chain as the research object, analyzes the Internet of Things (IoT) decisions and purchasing price decisions under different subsidy policies, and provides a reference for the government to select the appropriate type of subsidy policy. The literature closely related to this article mainly includes operation management of contract-farming supply chain, agricultural supply chain financing, and agricultural supply chain government subsidy.

(1) IoT applications

Agricultural production technologies based on the Internet of Things (IoT) offer several advantages, such as increasing production efficiency, enhancing product quality, and reducing production costs. These benefits are achieved through the application of advanced technologies like smart irrigation systems, intelligent temperature control systems, and remote monitoring and management systems, which optimize the growth process of agricultural products and improve their yield and quality. Adamo et al. presented an AI-powered microservices solution that optimizes irrigation and fertigation practices. The proposed system integrates IoT nodes for real-time data collection on environmental conditions, soil moisture levels, and nutrient crop needs [9]. This approach offers several benefits, including greater control over data flow, energy savings, and increased flexibility in resource management. Tan et al. [10] developed an IoT-based intelligent irrigation system for date palm orchards, which realizes intelligent remote control, real-time display, and alarm functions. An intelligent agricultural management system based on the Internet of Things (IoT) and cloud platforms can achieve remote monitoring and automated control of agricultural greenhouses. This not only effectively reduces labor management costs but also decreases energy consumption, thereby lowering the overall cost of agricultural production [11]. Chen et al. constructed an integrated “space-air-ground” IoT monitoring and control system, which strengthens the monitoring and application in potato production regarding soil moisture, field growth status, pest and disease conditions, and yield formation. This system realizes the automation, intelligence, and high efficiency of the digital agricultural production process in Anding District [12].The Jingshang Fruit and Vegetable Professional Cooperative in Weihai City covers an area of 94 hectares. The cooperative actively promotes the use of IoT technology. The person in charge highly endorses the integrated precision irrigation and fertilization technology. Statistics show that the use of chemical fertilizers has decreased by 15% compared to the same period, and water usage has been reduced by 83%. This has significantly lowered labor costs and improved management levels [13].The Smart Agriculture Industrial Park in Louxing District, Loudi City, Hunan Province, previously used traditional greenhouse planting methods. Under these methods, a couple could manage at most 2–3 greenhouses, with the output value per acre only being able to maintain at 10,000–20,000 yuan. However, now with digital management and production enabled by IoT technology, a couple can manage at least 10 greenhouses simultaneously within the park. The output value per acre has increased to 50,000–80,000 yuan, resulting in exponential growth in annual income [14].

(2) **Contract-farming supply chain operation management**

The research on the contract-farming supply chain mainly includes: the production and decision-making problems, the risk management problem, and the coordination problem. In the research on the production and decision-making of the contract-farming supply chain, Kazaz and Webster studied the production and pricing decisions of risk neutral and Risk aversion decision makers in the case of random output [15]. Ye and Wang studied the optimal output of agricultural products for a farmer and the optimal purchase price for a company under uncertain agricultural product output and sales influenced by output, and compared the optimal decision-making behavior under decentralized and centralized decision-making [16]. With the development of contract agriculture, the issue of default risk cannot be ignored. To solve this problem, scholars have proposed different methods. Fu and Dan found that adverse weather can exacerbate the risks faced by members in contract-farming during the performance process, and therefore designed order contracts based on the impact of weather [17]. Qin and Li considered the dual risks of demand and output uncertainty in the agricultural product market and proposed an order contract of “guaranteed minimum purchase, follow the market”, effectively reducing farmers’ risks [18]. In terms of contract-farming supply chain coordination, Yan et al. (2020)designed revenue sharing and wholesale price contracts for the fresh agricultural product newsboy model [19], while Pu and Yue coordinated the supply chain through revenue sharing contract and cost sharing contract [20]. The above literature has explored decision-making, risk management, and coordination issues in the contract-farming supply chain, but has not considered the impact of new technology application on supply chain operations. The agricultural IoT technology plays an important role in promoting the transformation of agriculture from extensive growth to technology driven growth [3]. In this context, this article considers the application of IoT technology in the contract-farming supply chain and studies the impact of IoT technology on the decision-making of contract-farming supply chain.

(3) **Research on agricultural supply chain financing**

The research on agricultural supply chain financing has also received widespread attention. Among them, some scholars confirmed that agricultural supply chain financing has increased farmers’ income. For example, Jiang and Wen demonstrated through empirical methods that agricultural order financing can help financially constrained scale farmers improve their profitability [21]. Wang et al. analyzed the investment of agricultural materials and the pricing of companies under financial constraint, and found that farmers can always improve their income through financing [22]. Lin et al. studied the decision of farmers constrained by funds when companies provide targeted and non targeted financing models, and found that trade financing can improve the profits of both farmers and companies [23]. In addition, many scholars have compared and analyzed different financing models, such as Ye et al., which fully considered output and demand risks and studied the returns of farmers under bank credit, trade credit, and combination credit [24]. Zhu et al. found that when the financing ability of farmers is weak, companies tend to provide financing services to farmers at lower interest rate. Moreover, when the company’s financing interest rate was within a certain range, the company’s financing model would achieve a win-win situation for both a company and a farmer [25]. Guo and Wang studied the performance of companies and farmers under the conditions of credit financing and trade financing based on the contract mechanism of “guaranteed buyout, follow the market”. They found that companies always hope that farmers choose financing methods with low expected returns, while farmers with high own funds prefer financing methods with high expected returns [26]. The above literature has studied the agricultural supply chain financing modes such as bank credit, trade credit and portfolio credit. Most of them focus on the financing of agricultural means of production, but lack the financing of agricultural production technology investment. Ma et al.establish a model to solve the equilibrium between bank financing and company targeted financing, and analyze the preferences of companies and farmers for different financing models [27]. Lu et al. examined three financing models in agricultural supply chains based on e-commerce advance orders: bank financing, e-commerce reverse factoring, and advance payment [28]. This paper introduces the Internet of Things (IoT) technology into the order agriculture supply chain and considers its bank financing model. It constructs decision-making models for the order agriculture supply chain under different government subsidy scenarios, exploring the effectiveness and schemes of government subsidies.

(4) **Research on government subsidy in agricultural supply Chain**

Agricultural subsidies are transfer payments made by the government to the production, distribution, and trade of agricultural products. They aim to support agricultural producers, stabilize the agricultural product market, ensure food security, and promote rural economic development. Interest rate subsidies and cost subsidies are two commonly used agricultural subsidy methods by the government. Cost subsidies refer to the direct financial support provided by the government to reduce the input costs of the farmer in the agricultural production process, such as the purchase of agricultural production materials and technical equipment. Interest rate subsidies, on the other hand, encourage production investment and technological upgrading by reducing the financing costs of the farmer.. Many scholars have studied the benefits of government subsidy, including the subsidy policy based on cultivated area [29], the yield subsidy policy [30], the cost subsidy policy for means of production [31], and the policy to reduce agricultural production taxes [32]. However, the above research only considered the impact of one subsidy policy on the agricultural supply chain, without comparing and analyzing the impact of different subsidy strategies on the decision-making of supply chain members. Comparative studies of multiple subsidy strategies, on the other hand, can uncover the substitutability between different policies, which is useful for policymakers to select more scientifically sound and rational policies. Wu et al. bases on e-commerce assistance to farmers and considers four types of government subsidies: no subsidy, price subsidy model with the farmer as the subsidy target, price subsidy model with the e-commerce platform as the subsidy target, and area subsidy model [33]. Lin et al.built a Stackelberg game decisionmaking model for a contract-farming supply chain under the weather index insurance framework to study the impacts of production cost and purchase price subsidies on each member of the supply chain.In addition, some studies have compared the effects of different government subsidy strategies through empirical methods [34]. For example, Zhao et al. used empirical research methods to find that the minimum purchase price policy for grain, agricultural machinery purchase subsidy policy, and fiscal land governance projects have significant promoting effects on China’s grain production [35]. Zhou et al. built a theoretical model of new classical economics and found that price subsidies, direct subsidies and agricultural machinery purchase subsidies are conducive to reducing the possibility of farmers’ part-time employment in non-agricultural sectors, stimulating farmers to engage in specialized agricultural production, promoting agricultural production increase and promoting agricultural production development [36]. However, these literature did not discuss which type of government subsidy can better promote the development of agricultural supply chains.

Previous literature has thoroughly examined agricultural supply chain operations, government subsidies, and financing mechanisms. However, limited research has explored the impact of Internet of Things (IoT) technology on agricultural production operations, as well as its implications for financing and government subsidies in this context. This study innovatively incorporates IoT technology into a contract farming supply chain, considering farmers’ financial constraints and bank financing. We develop decision-making models for contract farming supply chains under both subsidized and unsubsidized conditions, analyze the decisions and performance of supply chain stakeholders under different subsidy policies, and explore subsidy schemes under various government subsidy objectives.

It is important to emphasize that this study aims to construct a framework model to analyze the mechanisms by which agricultural subsidy policies impact decision-making and performance in contract farming supply chains. The focus is not on directly testing the effectiveness of specific policies in a particular context.

The strength of theoretical modeling lies in its ability to abstract complex systems to identify core variables and their interactions, thereby providing a foundational framework for future empirical research.

## 3. Model development

### 3.1 Problem description

**Fig 1:**
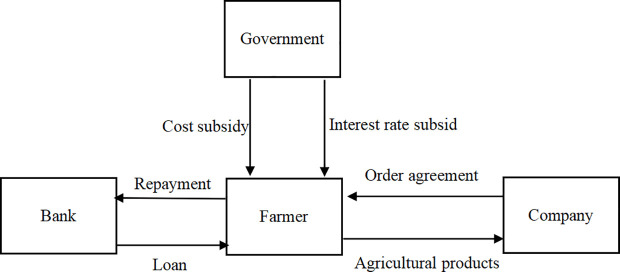
Structure of contract-farming supply chain under government subsidy.

This article takes the contract-farming supply chain composed of a farmer, a company, a government, and a bank as the research object, where the farmer have financial constraint. Before the agricultural production season, the company signs agricultural orders with the farmer at a certain price. The farmer is considering investing in the application of IoT technology to improve output efficiency. Due to the high investment in IoT technology, the farmer need to mortgage agricultural orders to obtain financing from a bank. The government encourages the farmer to apply IoT technology for environmental protection and food security reasons, and adopts two different subsidy policies, namely cost subsidy and interest rate subsidy. (As shown in Fig 1)

We divide agricultural production activities into pre production and post production. The order of the game is as follows: first, before production, the government announces subsidy policy and provides a certain proportion of subsidy. Subsequently, the bank provides IoT technology investment loans to the farmer. Next, the company sets the purchasing price. Then, the farmer determines the level of IoT application based on loan interest rate and subsidy. After the completion of production, the company purchases all agricultural products according to the contract, pays for the goods, and the government provides subsidies to the farmer. The subscript *f* represents the farmer, and the subscript *m* represents the company.

### 3.2 Symbol description

The symbols and their descriptions mainly used in this article are shown in Table 1.

**Table 1:** Related parameters.

Symbols	Description
$e_{i}$	The level of IoT adoption by the farmer (a decision variable of the farmer), i∈{0,1,2} where: i = 0 indicates no subsidy policy;i = 1 indicates an interest rate subsidy policy;i = 2 indicates a cost subsidy policy.
$w_{i}$	Buyer ‘s purchasing price of a agricultural product in case *i* (decision variable of the farmer), i∈{0,1,2}
q	Production without the application of IoT technology
a	Yield effect influenced by IoT technology
$c_{1}$	Unit agricultural material cost
$c_{2}$	Cost coefficient of the farmer effort
$c_{3}$	Cost coefficient of IoT investment
θ	The impact factor of IoT technology on reducing agricultural materials costs. By enabling precision fertilization through IoT, the farmer can reduce inputs of fertilizers, pesticides, and other agricultural supplies, thereby lowering input costs. The greater the value of θ, the more significant the reduction in agricultural materials costs achieved through IoT technology.
λ	The impact factor of IoT technology on reducing labor effort costs. By partially substituting human labor, IoT technology facilitates more convenient and intelligent agricultural production and management. The greater the value of λ, the more significant the reduction in labor effort costs achieved through IoT technology.
$r_{b}$	Bank rate, referring to the rate at which the farmer borrows funds from a bank.
p	Market price of agricultural products
v	Environmental friendliness, reflecting the impact of IoT adoption on the environment. Specifically, the farmer using IoT for precision fertilization can reduce the use of fertilizers, pesticides, and other inputs. The higher the value of v, the lower the environmental impact of agricultural production enabled by IoT.
$\pi_{fi}$	Farmer’s benefit, i∈{0,1,2}
$\pi_{mi}$	Company’s benefit, i∈{0,1,2}
$\pi_{bi}$	Bank’s benefit,i∈{0,1,2}
$SW_{i}$	Government’s benefit,i∈{0,1,2}

### 3.3 Hypothesis

(1) The level of IoT application is $e_{i}$,i∈{0,1,2}. According to the research of Ye et al. [24], the cost function is set to $c(e_{i})=c_{3}{e_{i}}^{2}$, where $c_{3}>0$ is the cost coefficient of the IoT.
(2) According to Wicaksono et al. [37], the output of crops by a farmer after applying IoT technology is $Q=qu_{\left(e_{i}\right)}$, where q is the output without IoT technology. $u=(1+ae_{i})$ is the coefficient of impact of IoT technology on yield, indicating that the higher the level of IoT application, the higher the yield of agricultural products.
(3) Referring to the research of Peng and Pang [29], Jirapond et al. [38], production costs are related to agricultural inputs and efforts such as seeds, fertilizers, and pesticides. In addition, applying IoT technology to accurately fertilize based on factors such as precipitation and air humidity, the farmer can reduce the amount of fertilizer and pesticide inputs, and reduce the cost of agricultural inputs. It is also possible to observe pests, crop growth, and other conditions in real-time through mobile terminals, thereby reducing the time, energy, and other costs invested by farmers. Therefore, the production cost function is set to $C_{\left(e_{i}\right)}=c_{1}q(1-\theta e_{i})+c_{2}q^{2}(1-\lambda e_{i})$. Where $c_{1}>0$ represents the unit agricultural material cost, including seeds, fertilizers, etc. θ>0 represents the impact factor of IoT technology on the reduction of agricultural material costs. $c_{2}>0$ represents the cost coefficient of farmers’ efforts. $c_{2}q^{2}$ represents the cost of production effort, including the time and energy consumed by farmers. λ>0 represents the impact factor of IoT technology on effort cost reduction.
(4) Before the production season, the initial capital of farmers is 0, and the farmer need to mortgage agricultural orders to obtain financing from the bank, and there is no possibility of bankruptcy for the farmer.
(5) The selling price of agricultural products is set as p, which is an exogenous variable and is greater than the purchasing price $p>w_{i}$ [7,22,30,34]. This ensures that the buyer remains profitable within the contract farming supply chain, which is also consistent with real-world scenarios.
(6) Both the farmer and the company are rational decision-makers, and both parties pursue the maximization of their own interests.

## 4. Subsidy policy

(1) **Case Ⅰ(Called no subsidy policy)**

In this scenario, the farmer applies for a loan $L_{0}$ with an interest rate of $r_{b}$ from the bank based on agricultural orders, with the loan principal and an interest being $L_{0}(1+r_{b})$. Where $L_{0}=c_{1}q(1-\theta e_{0})+c_{3}{e_{0}}^{2}$. The farmer’s decision objective function is


$$\pi_{f0}(e_{0})=w_{0}q(1+ae_{0})-[c_{1}q(1-\theta e_{0})+c_{3}{e_{0}}^{2}](1+r_{b})-c_{2}q^{2}(1-\lambda e_{0})$$ (1)

In the right part of Formula (1), the first item represents the total benefit obtained by the farmer applying IoT technology, the second item represents the production cost including agricultural materials cost, IoT input cost, and financing cost, and the third item represents the farmer’s effort cost.

The company’s decision objective function is


$$\pi_{m0}=(p-w_{0})q(1+ae_{0})$$ (2)

In the right part of Formula (2), $p-w_{0}$ represents marginal profit, $q(1+ae_{0})$ represents the output affected by the Internet of Things.

By backward induction, the optimal level of IoT application and the optimal purchasing price are obtained as follows:


$${e_{0}}^{*}=\frac{pqa^{2}+(ac_{1}q\theta -2c_{3})(1+r_{b})+ac_{2}\lambda q^{2}}{4ac_{3}(1+r_{b})}$$ (3)

$${w_{0}}^{*}=\frac{pqa^{2}-(ac_{1}q\theta +2c_{3})(1+r_{b})-ac_{2}\lambda q^{2}}{2qa^{2}}$$ (4)

**Proof**: Firstly, equation (1) can be used to derive:


$$\frac{d\pi_{f0}}{de_{0}}=aqw_{0}+(c_{1}q\theta -2c_{3}e_{0})(1+r_{b})+c_{2}\lambda q^{2}$$

$$\frac{d^{2}\pi_{f}}{d{e_{0}}^{2}}=-2c_{3}(1+r_{b})$$

So $\pi_{f0}$ is a strict concave function function with respect to $e_{0}$. Letting $\frac{d\pi_{m0}}{dw_{0}}=0$, we get the optimal application level of the IoT ${e_{0}}^{*}$. Then, by substituting ${e_{0}}^{*}$ into equation (2), we derive from equation (2):


$$\frac{d\pi_{m0}}{dw_{0}}=-(1+a{e_{0}}^{*})+a\frac{d{e_{0}}^{*}}{dw_{0}}(p-w_{0})$$

$$\frac{d^{2}\pi_{m0}}{d{w_{0}}^{2}}=-\frac{a^{2}q^{2}}{c_{3}(1+r_{b})}$$

Therefore, it is a strict concave function of $\pi_{m0}$ with respect to $w_{0}$. Letting $\frac{d\pi_{m0}}{dw_{0}}=0$, we can get the optimal purchase price ${w_{0}}^{*}$.

Proof completed.

Substituting equations (3) and (4) into equation (1), we can obtain the farmer’s benefit as follows:


$$\begin{array}{c} \pi_{f0}=\frac{pqa^{2}-(ac_{1}q\theta +2c_{3})(1+r_{b})-ac_{2}\lambda q^{2}}{2a^{2}}+\frac{{\left[pqa^{2}+(ac_{1}q\theta -2c_{3})(1+r_{b})+ac_{2}\lambda q^{2}\right]}^{2}}{16c_{3}a^{2}(1+r_{b})}\\ \;\;\;\;\;\;\;\;\;-c_{1}q(1+r_{b})-c_{2}q^{2} \end{array}$$ (5)

Substituting equations (3) and (4) into equation (2), we can obtain the company’s benefit as follows:


$$\pi_{m0}=\frac{{\left[pqa^{2}+(ac_{1}q\theta +2c_{3})(1+r_{b})+ac_{2}\lambda q^{2}\right]}^{2}}{8c_{3}a^{2}(1+r_{b})}$$ (6)

(2) **Case Ⅱ(Called interest rate subsidy policy)**

Under the interest rate subsidy policy, the government subsidizes the interest rate $r_{b}$ of IoT technology financing, with a subsidy rate of φ. And the farmer signs a contract with the company for a purchase price of $w_{1}$. Then the farmer borrows money from the bank based on the order, with an amount of $L_{1}$, where $L_{1}=c_{1}q(1-\theta e_{1})+c_{3}{e_{1}}^{2}$.

The farmer’s decision objective function is


$$\;\;\pi_{f1}(e_{1})=w_{1}q(1+ae_{1})-c_{1}q(1-\theta e_{1})(1+r_{b})-c_{2}q^{2}(1-\lambda e_{1})-c_{3}{e_{1}}^{2}[1+r_{b}(1-\varphi )]$$ (7)

In the right part of Formula (7), the first item represents the total benefit obtained by the farmer applying IoT technology, the second item represents the production cost including agricultural materials cost and financing cost, the third item represents the farmer’s effort cost, the forth item represents IoT input cost and financing cost.

The company’s decision objective function is


$$\pi_{m1}=(p-w_{1})q(1+ae_{1})$$ (8)

By backward induction, the optimal level of IoT application and the optimal purchasing price are obtained as follows:


$${e_{1}}^{*}=\frac{pqa^{2}+ac_{1}q\theta (1+r_{b})+ac_{2}\lambda q^{2}-2c_{3}[1+r_{b}(1-\varphi )]}{4ac_{3}[1+r_{b}(1-\varphi )]}$$ (9)

$${w_{1}}^{*}=\frac{pqa^{2}-ac_{1}q\theta (1+r_{b})-ac_{2}\lambda q^{2}-2c_{3}[1+r_{b}(1-\varphi )]}{2qa^{2}}$$ (10)

**Proof**: Firstly, differentiating equation (8) with respect to $e_{1}$, we have


$$\;\frac{\;d\pi_{f1}}{de_{1}}=w_{1}qa+c_{1}q\theta (1+r_{b})+c_{2}\lambda q^{2}-2c_{3}e_{1}[1+r_{b}(1-\varphi )]$$

$$\;\frac{\;d^{2}\pi_{f1}}{d{e_{1}}^{2}}=-2c_{3}[1+r_{b}(1-\varphi )]$$

From the above formula, we can see that $\pi_{f1}$ is a strict concave function function about $e_{1}$. Letting $\frac{d\pi_{f1}}{de_{1}}=0$, we can get under the case of interest rate subsidy policy, the optimal IoT application level ${e_{1}}^{*}=\frac{aqw_{1}+c_{1}q\theta (1+r_{b})+c_{2}\lambda q^{2}}{2c_{3}[1+r_{b}(1-\varphi )]}$.

Then, by substituting ${e_{1}}^{*}$ into equation (8), and taking the derivative of *w*_1_, we obtain


$$\frac{d\pi_{m1}}{dw_{1}}=-q(1+ae_{1})+\frac{q^{2}a^{2}(p-w_{1})}{2c_{3}[1+r_{b}(1-\varphi )]}$$

$$\frac{d^{2}\pi_{m1}}{d{w_{1}}^{2}}=-\frac{q^{2}a^{2}}{c_{3}[1+r_{b}(1-\varphi )]}$$

Therefore, it is a strict concave function of $\pi_{m1}$ with respect to $w_{1}$. Letting $\frac{d\pi_{m1}}{dw_{1}}=0$, we can get the optimal purchase price ${w_{1}}^{*}=\frac{pqa^{2}-ac_{1}q\theta (1+r_{b})-ac_{2}\lambda q^{2}-2c_{3}[1+r_{b}(1-\varphi )]}{2qa^{2}}$. Proof completed.

Substituting equations (9) and (10) into equation (7) and (8), the profits of the farmer and the company are obtained as follows:


$$\begin{array}{c} \pi_{f1}=\frac{pqa^{2}-ac_{1}q\theta (1+r_{b})-ac_{2}\lambda q^{2}-2c_{3}[1+r_{b}(1-\varphi )]}{2a^{2}}\\ +\frac{{\left\{pqa^{2}+ac_{1}q\theta (1+r_{b})+ac_{2}\lambda q^{2}-2c_{3}[1+r_{b}(1-\varphi )]\right\}}^{2}}{16c_{3}a^{2}[1+r_{b}(1-\varphi )]}\\ -c_{1}q(1+r_{b})-c_{2}q^{2} \end{array}$$ (11)

$$\pi_{m1}=\frac{{\left\{pqa^{2}+ac_{1}q\theta (1+r_{b})+ac_{2}\lambda q+2c_{3}[1+r_{b}(1-\varphi )]\right\}}^{2}}{8c_{3}a^{2}[1+r_{b}(1-\varphi )]}$$ (12)

(3) **Case Ⅲ(Called cost subsidy policy)**

Under the cost subsidy policy, the government provides subsidies to a farmer for the investment cost $c_{3}{e_{2}}^{2}$ of IoT technology with a subsidy rate of τ. The farmer signs a contract with the company for a purchase price of $w_{2}$, and the loan amount from the bank is $L_{2}$,where $L_{2}=c_{1}q(1-\theta e_{2})+c_{3}(1-\tau ){e_{2}}^{2}$.

The farmer’s decision objective function is


$$\;\;\pi_{f2}(e_{2})=w_{2}q(1+ae_{2})-[c_{1}q(1-\theta e_{2})+c_{3}(1-\tau ){e_{2}}^{2}](1+r_{b})-c_{2}q^{2}(1-\lambda e_{2})$$ (13)

In the right part of Formula (13), the first item represents the total benefit obtained by the farmer applying IoT technology, the second item represents the production cost including agricultural materials cost, IoT input cost, and financing cost, and the third item represents the farmer’s effort cost.

The company’s decision objective function is


$$\pi_{m2}=(p-w_{2})q(1+ae_{2})$$ (14)

Similarly, we obtain the optimal level of IoT application and the optimal purchasing price as follows:


$${e_{2}}^{*}=\frac{pqa^{2}+[ac_{1}q\theta -2c_{3}(1-\tau )](1+r_{b})+ac_{2}\lambda q^{2}}{4ac_{3}(1+r_{b})(1-\tau )}$$ (15)

$${w_{2}}^{*}=\frac{pqa^{2}-[ac_{1}q\theta +2c_{3}(1-\tau )](1+r_{b})-ac_{2}\lambda q^{2}}{2qa^{2}}$$ (16)

**Proof**:Firstly, differentiating equation (13) with respect to $e_{2}$, we have


$$\;\;\frac{d\pi_{f2}}{de_{2}}=w_{2}qa-[-c_{1}q\theta +2c_{3}e_{2}(1-\tau )](1+r_{b})+c_{2}\lambda q^{2}$$

$$\;\;\frac{d^{2}\pi_{f2}}{d{e_{2}}^{2}}=-2c_{3}(1-\tau )(1+r_{b})$$

We can find that $\pi_{f2}$ is a strict concave function function about $e_{2}$. Letting $\frac{d\pi_{f2}}{de_{2}}=0$, we can get under the case of interest rate subsidy policy, the optimal IoT application level ${e_{2}}^{*}=\frac{aqw_{2}+c_{1}\theta q(1+r_{b})+c_{2}\lambda q^{2}}{2c_{3}(1+r_{b})(1-\tau )}$.

And, by substituting ${e_{2}}^{*}$ into equation (13), and taking the derivative of *w*_2_, we obtain


$$\frac{d\pi_{m2}}{dw_{2}}=-q(1+ae_{2})+(p-w_{2})\frac{q^{2}a^{2}}{2c_{3}(1+r_{b})(1-\tau )}$$

$$\frac{d\pi_{m2}}{dw_{2}}=-\frac{q^{2}a^{2}}{c_{3}(1+r_{b})(1-\tau )}$$

Therefore, it is a strict concave function of $\pi_{m2}$ with respect to $w_{2}$. Letting $\frac{d\pi_{m2}}{dw_{2}}=0$, we can get the optimal purchase price ${w_{2}}^{*}$. Proof completed.

Substituting equations (15) and (16) into equation (13) and (14), the profits of the farmer and the company are obtained as follows:


$$\begin{array}{c} \pi_{f2}=\frac{pqa^{2}-[ac_{1}q\theta +2c_{3}(1-\tau )](1+r_{b})-ac_{2}\lambda q^{2}}{2a^{2}}\\ +\frac{{\left\{pqa^{2}+[ac_{1}q\theta -2c_{3}(1-\tau )](1+r_{b})+ac_{2}\lambda q^{2}\right\}}^{2}}{16c_{3}a^{2}(1+r_{b})(1-\tau )}\\ -c_{1}q(1+r_{b})-c_{2}q^{2} \end{array}$$ (17)

$$\pi_{m2}=\frac{{\left\{pqa^{2}+[ac_{1}q\theta +2(1-\tau )c_{3}](1+r_{b})+ac_{2}\lambda q^{2}\right\}}^{2}}{8c_{3}a^{2}(1+r_{b})(1-\tau )}$$ (18)

As can be seen from the above, under no subsidy policy, cost subsidy policy, and interest rate subsidy policy, the optimal decisions and benefits of the farmer and the company are all related to parameters such as a、λ、θ. Next, we will analyze the impact of these parameters on the farmer’s and company’s decisions, as well as their benefits.

## 5. Discussion and analysis

(1) **Analysis of decision variables on parameters**

Firstly, we analyze the impact of bank interest rate on the optimal decisions and benefits of the farmer and the company in the above three scenarios, and obtain inference 1.

**Inference 1:** There is a relationship between bank interest rate and the decision variables and benefits of contract-farming supply chain:

$\frac{d{e_{0}}^{*}}{dr_{b}}<0$,$\frac{d{e_{1}}^{*}}{dr_{b}}<0$,$\frac{d{e_{2}}^{*}}{dr_{b}}<0$;$\frac{d{w_{0}}^{*}}{dr_{b}}<0$,$\frac{d{w_{1}}^{*}}{dr_{b}}<0$,$\frac{d{w_{2}}^{*}}{dr_{b}}<0$;$\frac{d\pi_{f0}}{dr_{b}}<0$,$\frac{d\pi_{f1}}{dr_{b}}<0$,$\frac{d\pi_{f2}}{dr_{b}}<0$;$\frac{d\pi_{m0}}{dr_{b}}<0$,$\frac{d\pi_{m1}}{dr_{b}}<0$,$\frac{d\pi_{m2}}{dr_{b}}<0$.

Then, we will consider further validating the impact of bank interest rate on the optimal decisions and benefits of the farmer and the company through numerical simulations. The relevant parameter variables are set as follows:

a=0.1,θ=0.3,λ=0.2,q=10000,p=4,$c_{1}=0.1$,$c_{2}=0.00001$,$c_{3}=100$,φ=0.3,τ=0.02,$r_{b}=[0,0.1]$.

**Fig 2:**
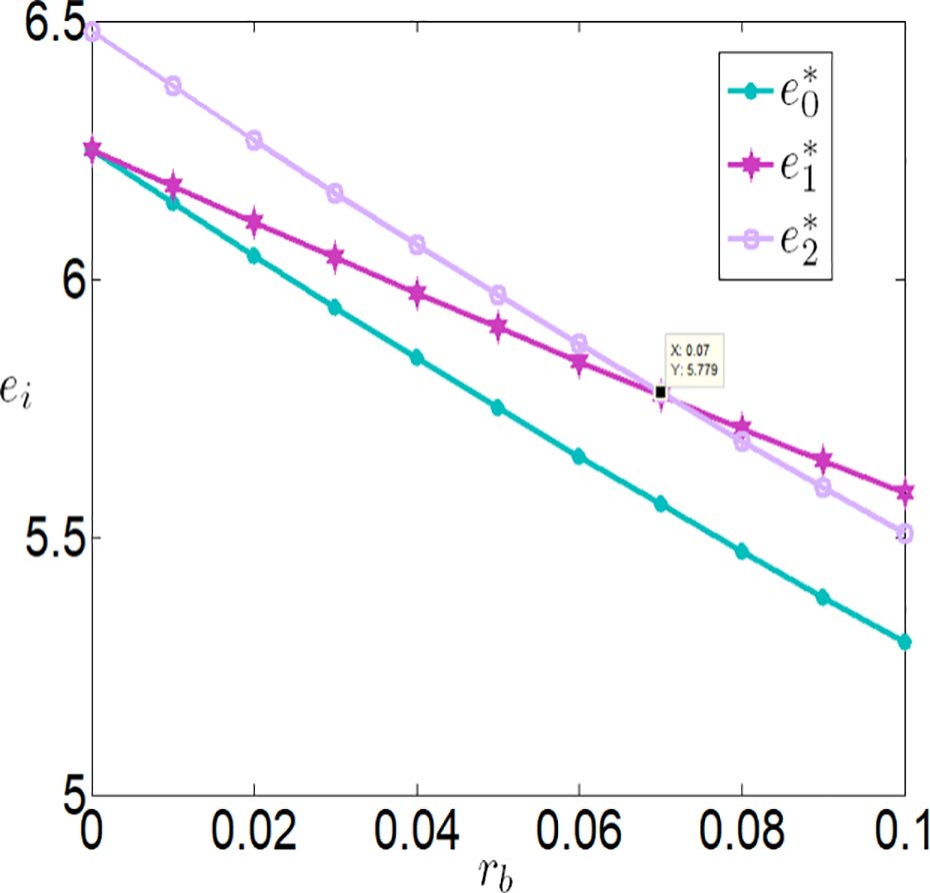
The impact of $\begin{array}{c} r_{b} \end{array}$ on $\begin{array}{c} e_{i} \end{array}$.

**Fig 3:**
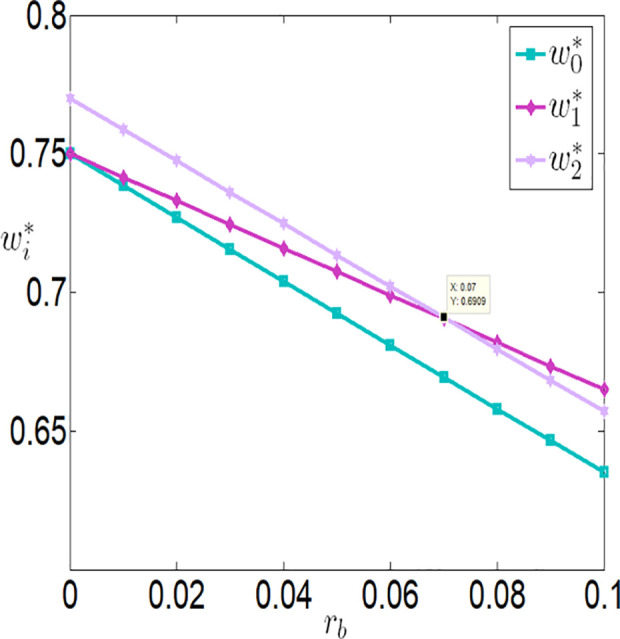
The impact of $\begin{array}{c} r_{b} \end{array}$ on $\begin{array}{c} w_{i} \end{array}$.

**Fig 4:**
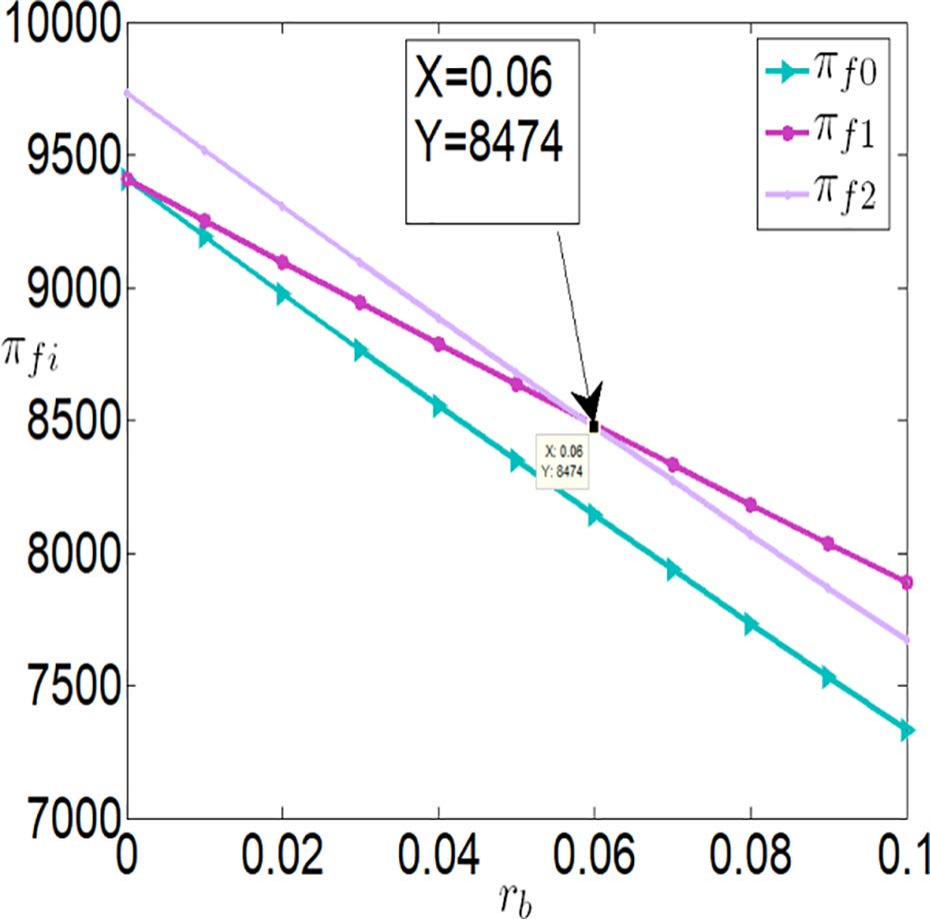
The impact of $\begin{array}{c} r_{b} \end{array}$ on $\begin{array}{c} \pi_{fi} \end{array}$.

**Fig 5:**
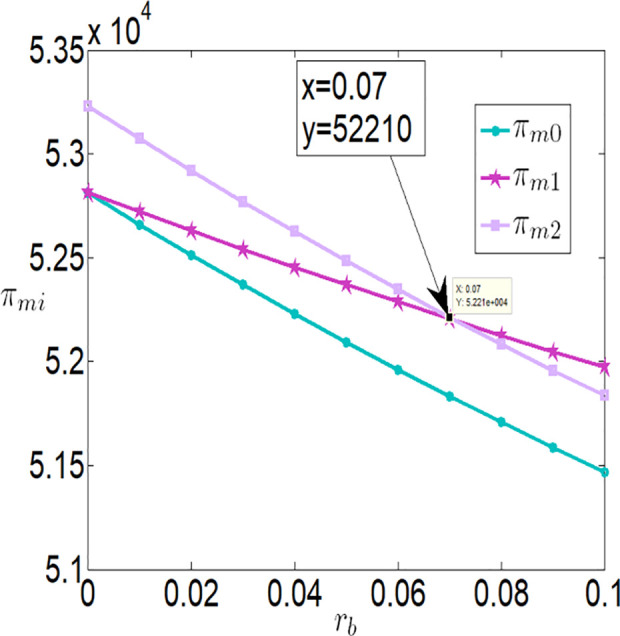
The impact of $\begin{array}{c} r_{b} \end{array}$ on $\begin{array}{c} \pi_{mi} \end{array}$.

As shown in Figs 2–5, irrespective of whether the farmer benefits from a subsidy policy, the application level of the Internet of Things (IoT), the purchase price, the farmer’s profit, and the buyer’s profit are all inversely proportional to the interest rate. The key reasons are as follows:

1. **Higher financing costs reduce the farmer’s profit**: As the interest rate increases, the financing costs paid by the farmer rise, which directly lowers the farmer’s profit. To address this, the farmer may adopt strategies to balance IoT investments and financing, such as reducing investments in IoT to safeguard their returns.
2. **Adverse effects on the supply chain**: While banks gain higher profits as financing rates increase, both the farmer and the buyer experience reduced profits. This is because higher financing rates lower the application level of IoT, which negatively impacts agricultural output. With market prices held constant, the buyer must lower the purchase price to increase marginal profits and offset losses from insufficient sales volume. Thus, when interest rates rise, the farmer faces dual adverse effects from lower purchase prices and reduced output.

Furthermore, Figs 2–5 indicates that when the financing rate exceeds a certain threshold, the farmer is more inclined to prefer an interest rate subsidy policy over a cost subsidy policy. Specifically:

When the financing rate exceeds 0.6, the farmer’s profit under an interest rate subsidy policy is also higher than that under a cost subsidy policy.

When the financing rate exceeds 0.7, the IoT application level, purchase price, and buyer’s profit under an interest rate subsidy policy are higher than those under a cost subsidy policy.

Second, by analyzing the yield effect a on the optimal decisions and profits of the farmer and the buyer, we derive Inference 2.

Inference 2: The yield effect of Internet of Things (IoT) technology has the following relationship with the decisions and performance of the contract farming supply chain.

$\frac{d{e_{0}}^{*}}{da}>0$,$\frac{d{e_{1}}^{*}}{da}>0$,$\frac{d{e_{2}}^{*}}{da}>0$;$\frac{d{w_{0}}^{*}}{da}>0$,$\frac{d{w_{1}}^{*}}{da}>0$,$\frac{d{w_{2}}^{*}}{da}>0$;$\frac{d\pi_{f0}}{da}>0$,$\frac{d\pi_{f1}}{da}>0$,$\frac{d\pi_{f2}}{da}>0$;$\frac{d\pi_{m0}}{da}>0$,$\frac{d\pi_{m1}}{da}>0$,$\frac{d\pi_{m2}}{da}>0$.

We will consider further validating the impact of the yield effect a on the optimal decisions and benefits of the farmer and the buyer through numerical simulations. The relevant parameter variables are set as follows:

θ=0.3, λ=0.2, q=10000, p=4, $c_{1}=0.1$, $c_{2}=0.00001$, $c_{3}=100$, $r_{b}=0.03$, φ=0.3, τ=0.02, a=[0.09,0.1].

**Fig 6:**
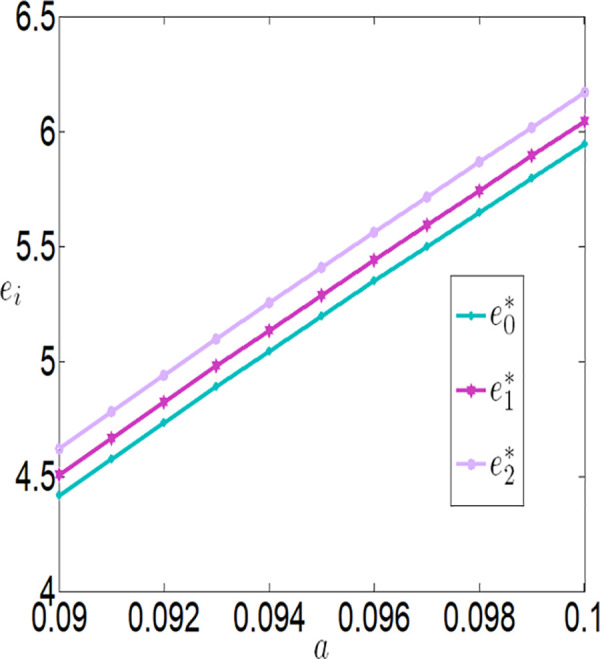
The impact of $\begin{array}{c} a \end{array}$ on $\begin{array}{c} e_{i} \end{array}$.

**Fig 7:**
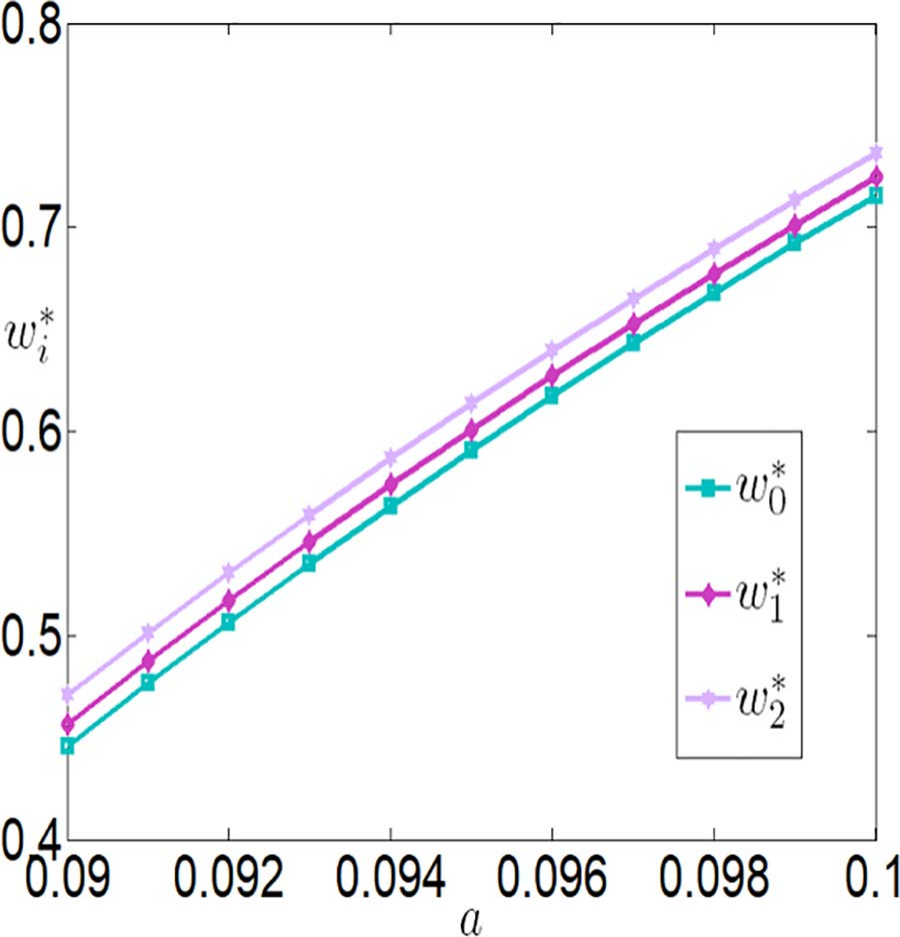
The impact of $\begin{array}{c} a \end{array}$ on $\begin{array}{c} w_{i} \end{array}$.

**Fig 8:**
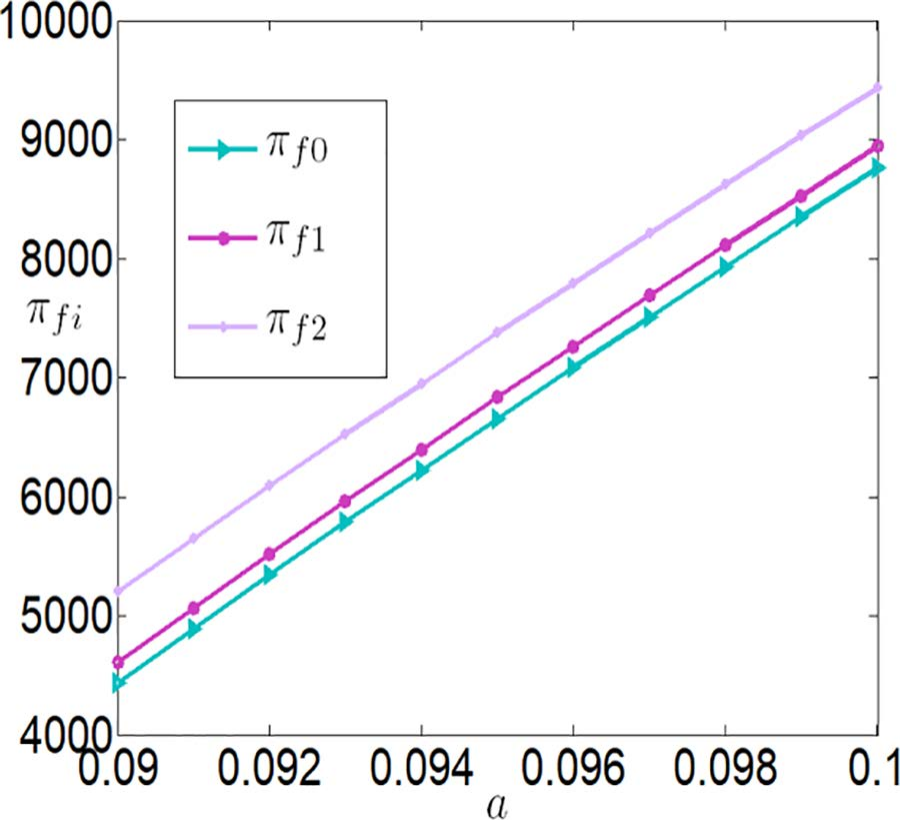
The impact of $\begin{array}{c} a \end{array}$ on $\begin{array}{c} \pi_{fi} \end{array}$.

**Fig 9:**
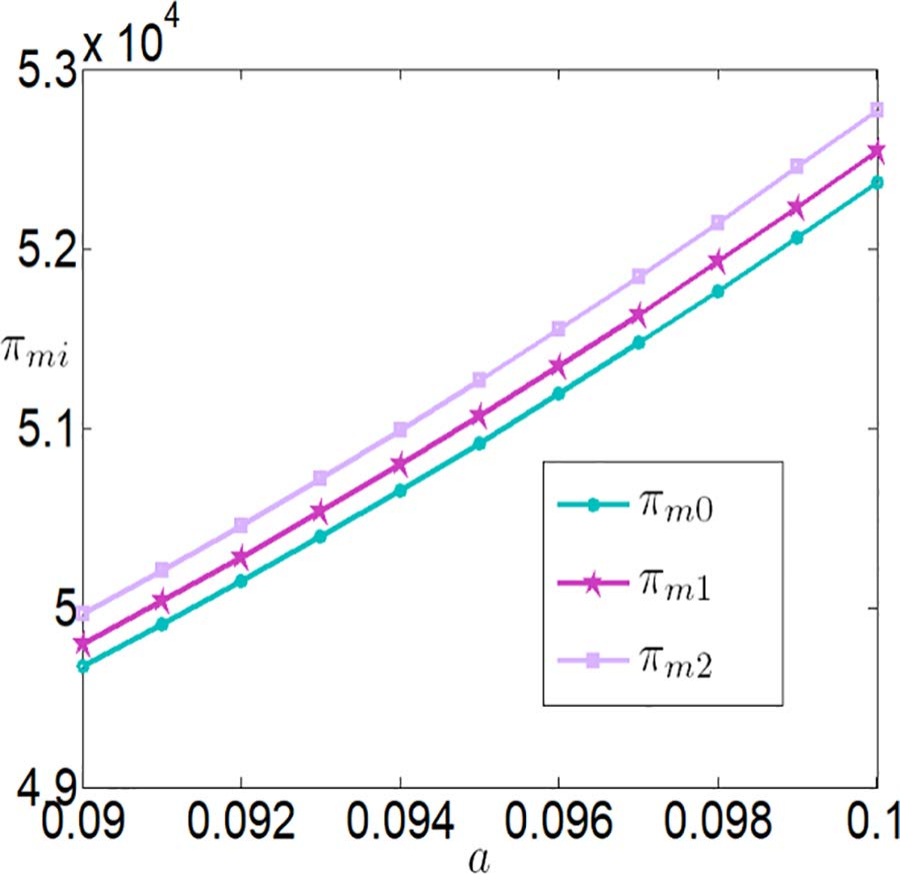
The impact of $\begin{array}{c} a \end{array}$ on $\begin{array}{c} \pi_{mi} \end{array}$.

As shown in Figs 6–9, under different subsidy policies, the application level of the Internet of Things (IoT), purchase prices, and the profits of both the farmer and the buyer are positively correlated with a. As the yield-enhancing effects of IoT technology become more pronounced, the farmer’s willingness to adopt IoT technology also intensifies. The application level of IoT technology not only increases production but also creates a ripple effect—purchase prices rise, and the farmer’s profit improves significantly. The buyer increases the purchase price of agricultural products because it incentivizes the farmer to adopt IoT technology and boost production, thereby generating higher sales profits.

Thirdly, we analyze the impact of effect factor θ on the optimal decisions and benefits of supply chain, and obtain inference 3.

**Inference 3:** The cost-reduction effect of IoT technology on agricultural production materials is correlated with the decisions and performance of the contract-farming agricultural supply chain:

$\frac{d{e_{0}}^{*}}{d\theta }>0$,$\frac{d{e_{1}}^{*}}{d\theta }>0$,$\frac{d{e_{2}}^{*}}{d\theta }>0$;$\frac{d{w_{0}}^{*}}{d\theta }<0$,$\frac{d{w_{1}}^{*}}{d\theta }<0$,$\frac{d{w_{2}}^{*}}{d\theta }<0$;$\frac{d\pi_{m0}}{d\theta }>0$,$\frac{d\pi_{m1}}{d\theta }>0$,$\frac{d\pi_{m2}}{d\theta }>0$.

Under no subsidy policy, if $c_{3}<\frac{pqa^{2}+ac_{1}q\theta (1+r_{b})+ac_{2}\lambda q^{2}}{6(1+r_{b})}$, then $\frac{d\pi_{f0}}{d\theta }>0$, and vice versa;

Under interest rate subsidy policy, if $c_{3}<\frac{pqa^{2}+ac_{1}q\theta (1+r_{b})+ac_{2}\lambda q^{2}}{6[1+r_{b}(1-\varphi )]}$, then $\frac{d\pi_{f1}}{d\theta }>0$, and vice versa;

Under cost subsidy policy, if $c_{3}<\frac{pqa^{2}+ac_{1}q\theta (1+r_{b})+ac_{2}\lambda q^{2}}{6(1-\tau )(1+r_{b})}$,then $\frac{d\pi_{f2}}{d\theta }>0$,and vice versa.

Similarly, we have a=0.1, λ=0.2, q=10000, p=4, $c_{1}=0.1$, $c_{2}=0.00001$, $c_{3}=100$, $r_{b}=0.03$, φ=0.3, τ=0.02, θ∈[0,0.5].

Fourthly, we analyze the impact of effect factor λ on the optimal decisions and benefits of supply chain, and obtain inference 4.

**Inference 4:** The cost effect of IoT technology efforts is related to the decisions and benefits of contract-farming supply chain as follows:

$\frac{d{e_{0}}^{*}}{d\lambda }>0$,$\frac{d{e_{2}}^{*}}{d\lambda }>0$,$\frac{d{e_{1}}^{*}}{d\lambda }>0$;$\frac{d{w_{0}}^{*}}{d\lambda }<0$,$\frac{d{w_{1}}^{*}}{d\lambda }<0$,$\frac{d{w_{2}}^{*}}{d\lambda }<0$;$\frac{d\pi_{m0}}{d\lambda }>0$,$\frac{d\pi_{m1}}{d\lambda }>0$,$\frac{d\pi_{m2}}{d\lambda }>0$;

Under no subsidy policy, if $c_{3}<\frac{pqa^{2}+ac_{1}q\theta (1+r_{b})+ac_{2}\lambda q^{2}}{6(1+r_{b})}$, then $\frac{d\pi_{f0}}{d\lambda }>0$, and vice versa;

Under interest rate subsidy policy,if $c_{3}<\frac{pqa^{2}+ac_{1}q\theta (1+r_{b})+ac_{2}\lambda q^{2}}{6[1+r_{b}(1-\varphi )]}$, then $\frac{d\pi_{f1}}{d\lambda }>0$, and vice versa;

Under cost subsidy policy, if$c_{3}<\frac{pqa^{2}+(ac_{1}q\theta )(1+r_{b})+ac_{2}\lambda q^{2}}{6(1-\tau )(1+r_{b})}$,then $\frac{d\pi_{f2}}{d\lambda }>0$, and vice versa.

Similarly, we have a=0.1, θ=0.3, q=10000, p=4, $c_{1}=0.1$, $c_{2}=0.00001$, $r_{b}=0.03$, φ=0.3, τ=0.02.

**Fig 10:**
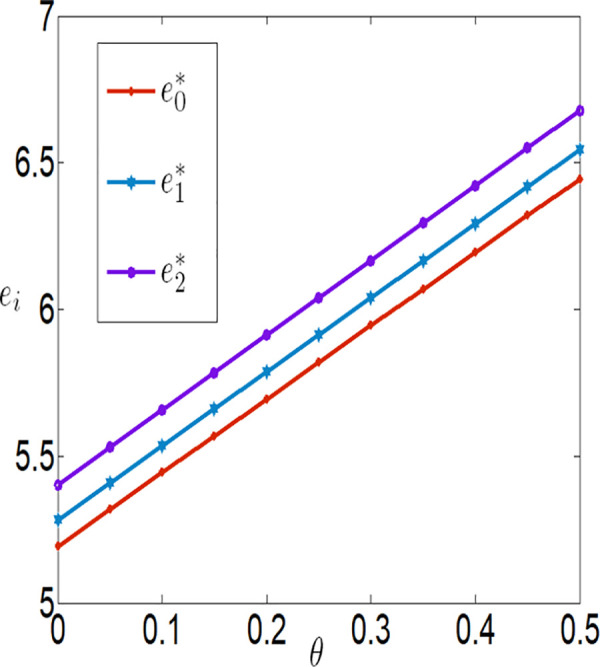
The impact of $\begin{array}{c} \theta \end{array}$ on $\begin{array}{c} e_{i} \end{array}$.

**Fig 11:**
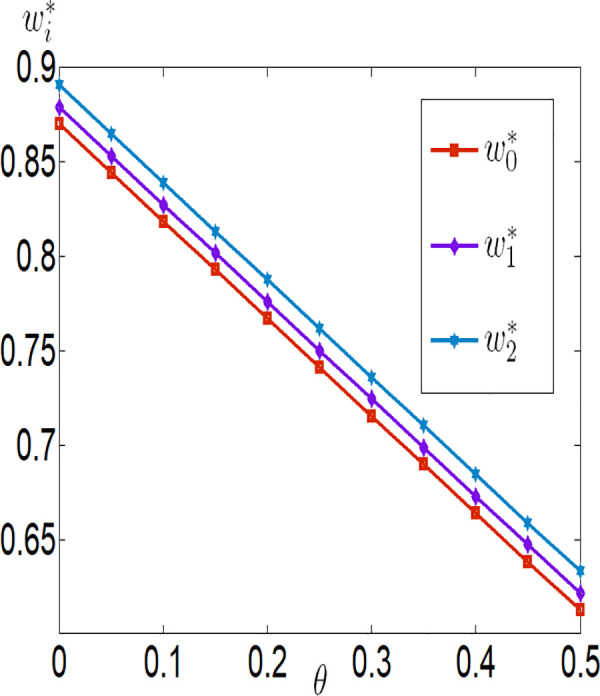
The impact of $\begin{array}{c} \theta \end{array}$ on $\begin{array}{c} w_{i} \end{array}$.

**Fig 12:**
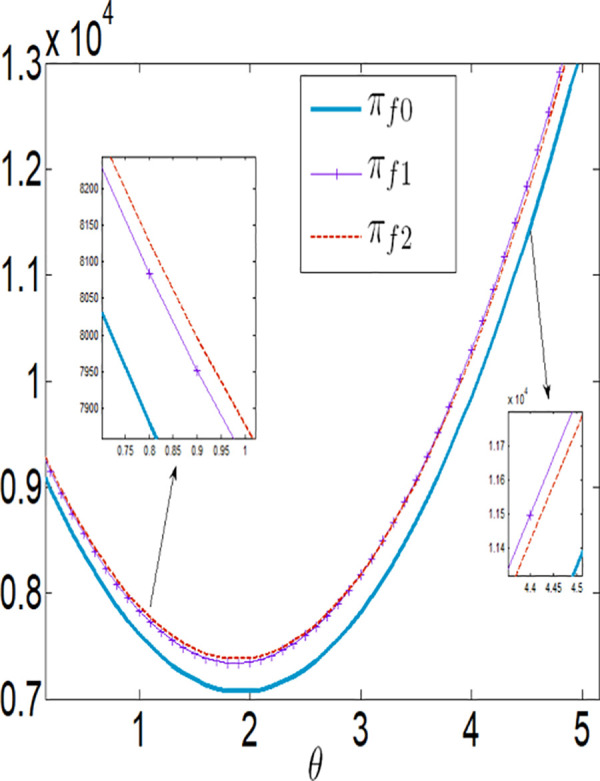
The impact of $\begin{array}{c} \theta \end{array}$ on $\begin{array}{c} \pi_{fi} \end{array}$.

**Fig 13:**
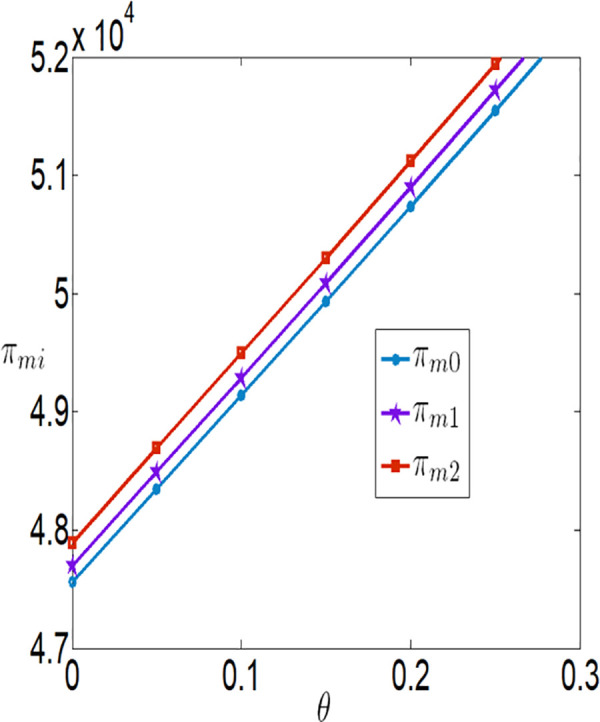
The impact of $\begin{array}{c} \theta \end{array}$ on $\begin{array}{c} \pi_{mi} \end{array}$.

**Fig 14:**
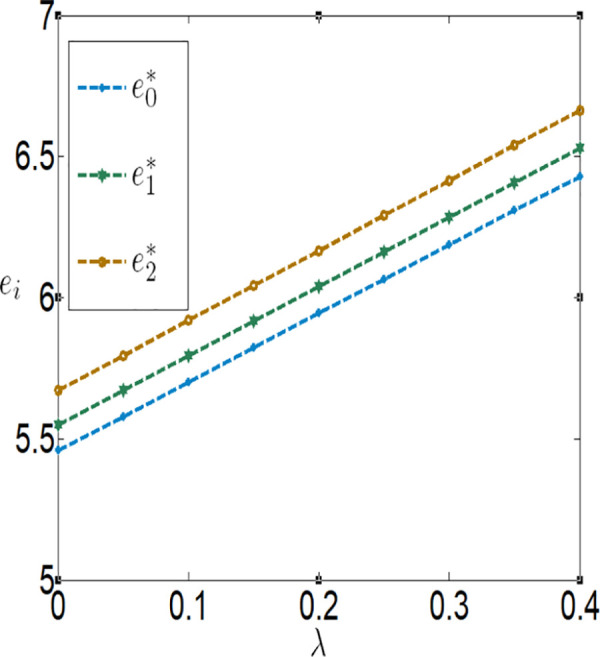
The impact of $\begin{array}{c} \lambda \end{array}$ on $\begin{array}{c} e_{i} \end{array}$.

**Fig 15:**
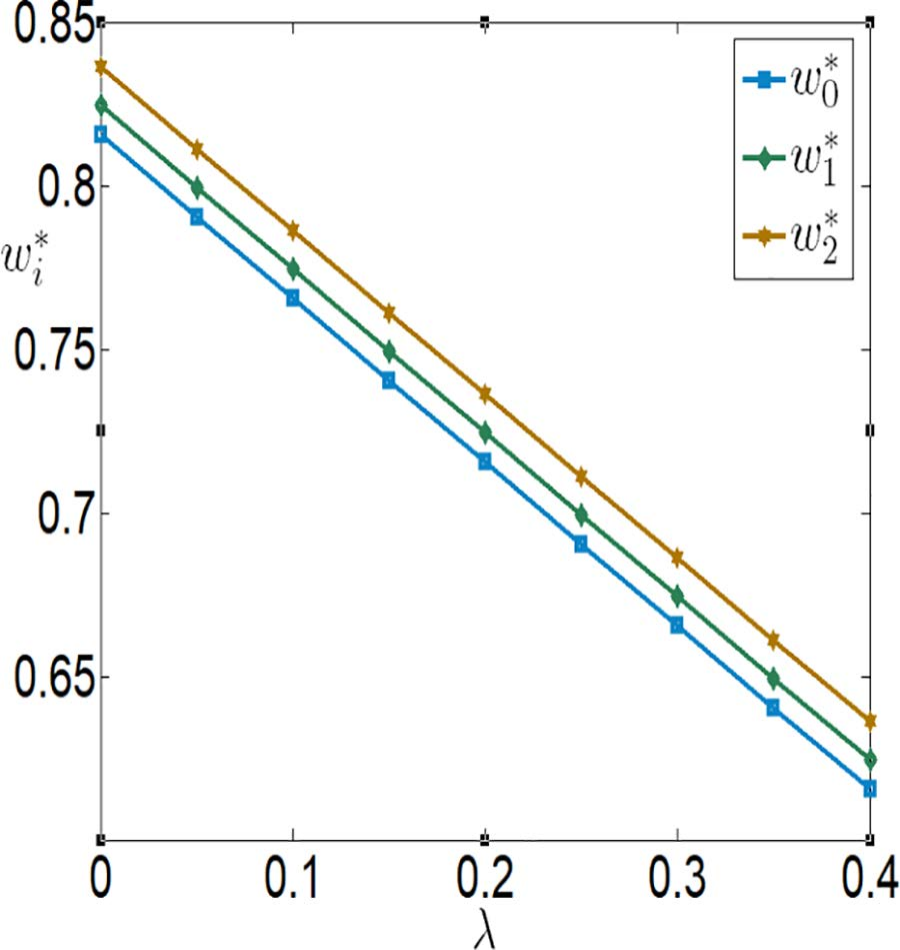
The impact of $\begin{array}{c} \lambda \end{array}$ on $\begin{array}{c} w_{i} \end{array}$.

**Fig 16:**
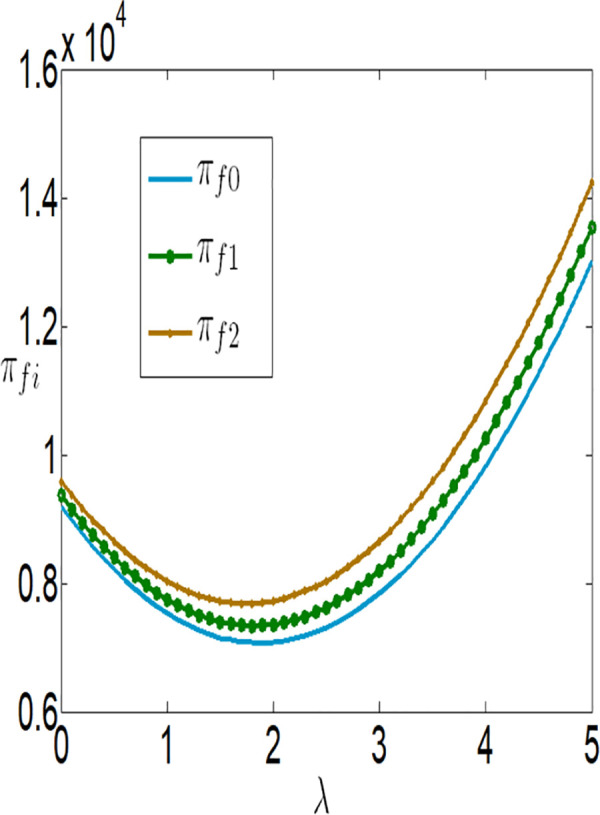
The impact of $\begin{array}{c} \lambda \end{array}$ on $\begin{array}{c} \pi_{fi} \end{array}$.

**Fig 17:**
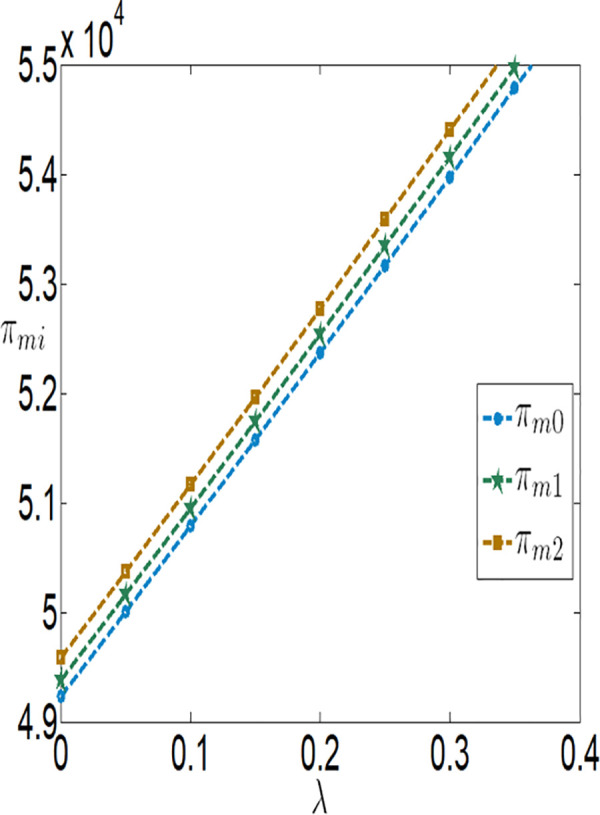
The impact of $\begin{array}{c} \lambda \end{array}$ on $\begin{array}{c} \pi_{mi} \end{array}$.

Figs 10–17 indicate that, regardless of whether subsidies are in place, the greater the impact of the Internet of Things (IoT) on reducing the costs of agricultural inputs and labor, the higher the level of IoT technology adoption, and the more motivated the farmer is to invest in IoT. Conversely, the lower the impact, the less motivated the farmer is to invest. When the positive impact of IoT on reducing these costs is small, the use of IoT can actually increase the burden on the farmer. In other words, the benefits generated by IoT are insufficient to offset the costs incurred. However, when the positive impact of IoT on reducing these costs is large, the use of IoT can bring positive returns to the farmer, who will then be more inclined to invest in IoT. Therefore, it can be observed that the farmer’s profit first decreases and then increases with the increase in λ and θ. In addition, the company’s purchase price decreases with the increase in λ and θ, while the company’s profit increases with the increase in λ and θ.

Fifthly, we analyze the impact of cost coefficient of IoT investment $c_{3}$ on the optimal decisions and benefits of supply chain, and obtain inference 5.

**Inference 5:** The cost coefficient of IoT investment is related to the decisions and benefits of contract-farming supply chain as follows:

$\frac{d{e_{0}}^{*}}{dc_{3}}<0$,$\frac{d{e_{1}}^{*}}{dc_{3}}<0$,$\frac{d{e_{2}}^{*}}{dc_{3}}<0$;$\frac{d{w_{0}}^{*}}{dc_{3}}<0$,$\frac{d{w_{1}}^{*}}{dc_{3}}<0$,$\frac{d{w_{2}}^{*}}{dc_{3}}<0$,$\frac{d\pi_{f0}}{dc_{3}}<0$,$\frac{d\pi_{f1}}{dc_{3}}<0$,$\frac{d\pi_{f2}}{dc_{3}}<0$;$\frac{d\pi_{m0}}{dc_{3}}<0$.$\frac{d\pi_{m1}}{dc_{3}}<0$,$\frac{d\pi_{m2}}{dc_{3}}<0$.

Inference 5 indicates that under different subsidy policies, the application level of Internet of Things (IoT) technology, purchase prices of agricultural products, and the profits of both the farmer and the buyer all decrease with rising IoT application costs. Specifically, higher IoT costs lower purchase prices, dampening the farmer’s enthusiasm for adopting the technology. The buyer’s profit declines due to reduced production lowering sales revenue; even though purchase prices drop, it is not enough to offset this loss.

Sixthly, we analyze the impact of subsidy rate on the optimal decisions and benefits of supply chain, and obtain inference 6.

**Inference 6:** The subsidy rate for financing interest rate is related to the decisions and benefit of contract-farming supply chain as follows:

$\frac{d{e_{1}}^{*}}{d\varphi }>0$,$\frac{d{w_{1}}^{*}}{d\varphi }>0$,$\frac{d\pi_{f1}}{d\varphi }>0$,$\frac{d\pi_{m1}}{d\varphi }>0$.

Inference 6 shows that government interest rate subsidies can motivate the farmer to adopt IoT technology, thereby increasing the profits of both the farmer and the buyer. However, unexpectedly, this subsidy indirectly raises the purchase price of agricultural products. This occurs because, with stable market prices, an increase in the sales volume of agricultural products necessarily boosts the buyer’s profits. Therefore, to obtain more agricultural products and earn higher profits, the buyer raises the purchase price, encouraging the farmer to more widely apply IoT technology.

Finally, we analyze the impact of cost subsidy rate on the optimal decisions and benefits of supply chain, and obtain inference 7.

**Inference 7:** The cost subsidy rate is related to the decisions and benefit of contract-farming supply chain as follows:$\frac{d{e_{2}}^{*}}{d\tau }>0$,$\frac{d{w_{2}}^{*}}{d\tau }>0$,$\frac{d\pi_{f2}}{d\tau }>0$,$\frac{d\pi_{m2}}{d\tau }>0$.

(2) Analysis of the Effectiveness of Government Subsidy Policies

The purpose of government subsidies for farmers’ application of Internet of Things (IoT) technology is to enhance the level of IoT application among farmers, increase their income, and achieve social benefits. To compare the effectiveness of two types of government subsidies, it is necessary to address the comparability of government subsidies. Assuming that the government has the same subsidy expenditure as a precondition, the effectiveness of different government subsidy policies is analyzed and compared.

The expenditures for government interest rate subsidies and cost subsidies are as follows, respectively:


$$SG_{\left(\varphi \right)}=\varphi r_{b}c_{3}{e_{1}}^{2}$$ (19)

$$SG_{\left(\tau \right)}=\tau c_{3}{e_{2}}^{2}$$ (20)

By combining equations (19) and (20), it can be seen that as the government subsidy coefficients φ and τ increase, the government’s subsidy expenditure also increases. When the government subsidy expenditures under the two subsidy mechanisms are equal, that is, when equations (19) and (20) are equal ($Z=SG_{\left(\varphi \right)}=SG_{\left(\tau \right)}$), we can derive the relationship between φ and τ, as shown below.


$$\begin{array}{c} \varphi r_{b}\{\frac{pqa^{2}+ac_{1}q\theta (1+r_{b})+ac_{2}\lambda q^{2}-2[1+r_{b}(1-\varphi )]c_{3}}{4ac_{3}[1+r_{b}(1-\varphi )]}{\}}^{2}\\ =\tau \{\frac{pqa^{2}+ac_{1}q\theta (1+r_{b})+ac_{2}\lambda q^{2}-2c_{3}(1-\tau )(1+r_{b})}{4ac_{3}(1+r_{b})(1-\tau )}{\}}^{2} \end{array}$$ (21)

Where, Z represents the government subsidy expenditure._。_

To quantify the social benefit value, this paper draws on the research of Krass et al. [39] and Cao et al. [40], and constructs the social benefit function as a composite of five components: the income of the farmer, the profit of the agricultural product buyer, the profit of the bank, government expenditure, and environmental benefits. As shown below.


$$SW_{i}=\pi_{fi}+\pi_{mi}+\pi_{bi}-SG_{i}+SE_{i}$$ (22)

Here, $SE_{i}$ represents the social benefit function, indicating the positive impact on the social environment after the application of Internet of Things (IoT) technology, that is, $SE_{i}=ve_{i}$.v denotes the environmental friendliness factor coefficient. i=1 represents the interest rate subsidy policy, while i=2 represents the cost subsidy policy.

Next, we consider using numerical simulation to analyze the differences in IoT application levels, supply chain member performance, and social welfare under the two different subsidy policies. We seta=0.1, θ=0.3, λ=0.2, q=10000, p=4, $c_{1}=0.1$, $c_{2}=0.00001$, $c_{3}=100$, $r_{b}=0.03$, φ=[0,0.7]. We vary the interest rate subsidy ratio within the range φ=[0,0.7] with a step size of 0.1. This allows us to obtain the cost subsidy coefficients and government expenditure under the same level of government fiscal subsidy expenditure (see Table 2).

**Table 2:** Corresponding values of the two subsidy coefficients and government expenditure when the government subsidy expenditure is the same.

φ	0.1	0.2	0.3	0.4	0.5	0.6	0.7
τ	0.003	0.006	0.009	0.012	0.015	0.018	0.021
*Z*	10.714	21.660	32.840	44.260	55.923	67.834	79.995

**Fig 18:**
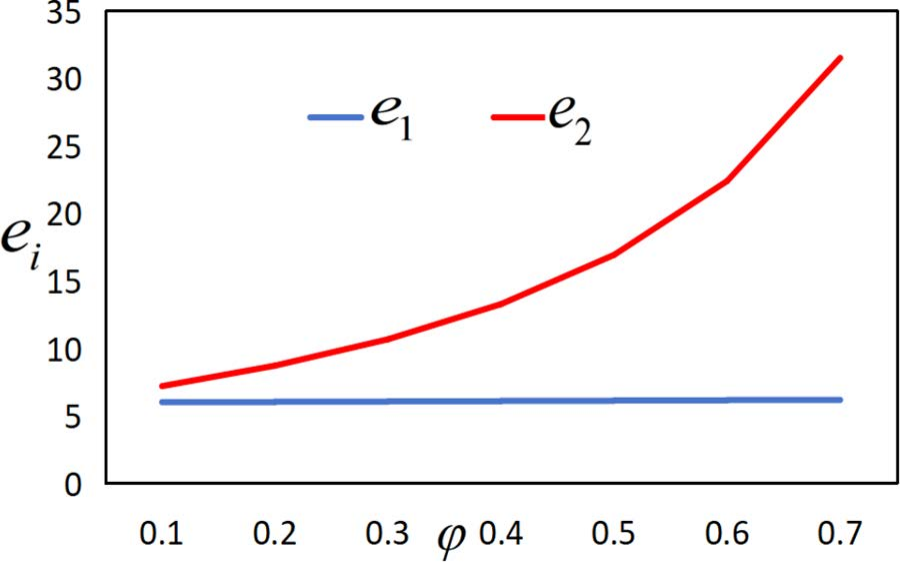
The impact of $\begin{array}{c} \varphi \end{array}$ on $\begin{array}{c} e_{i} \end{array}$.

**Fig 19:**
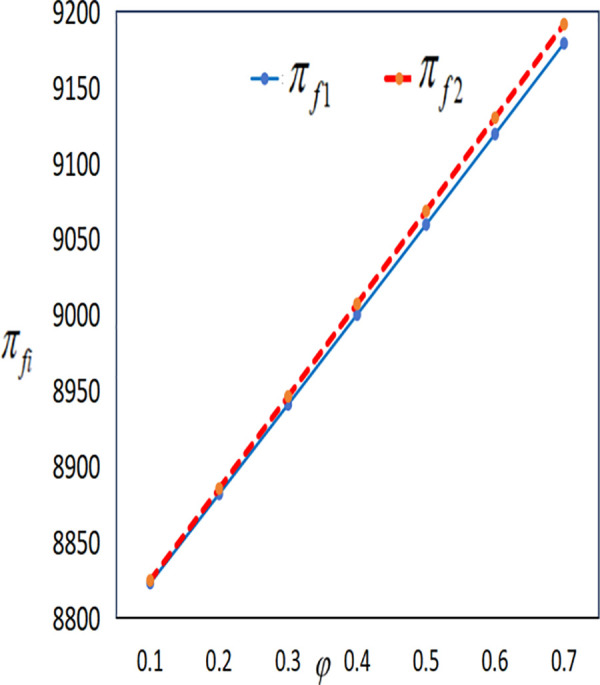
The impact of $\begin{array}{c} \varphi \end{array}$ on $\begin{array}{c} \pi_{fi} \end{array}$.

**Fig 20:**
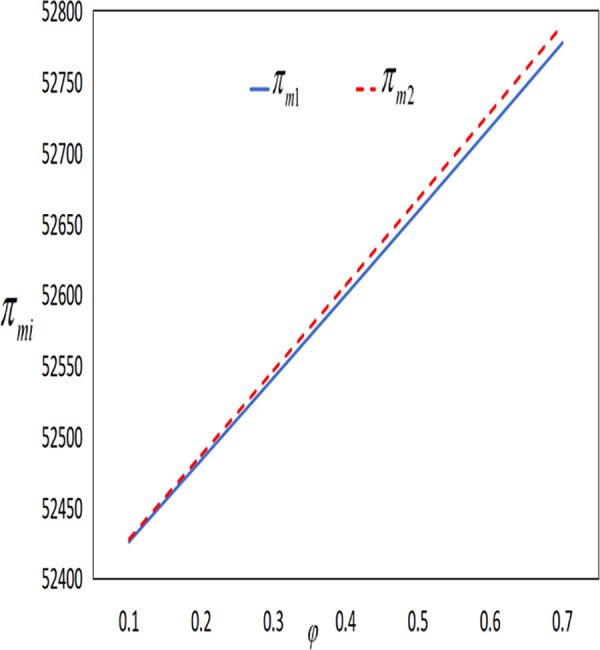
The impact of $\begin{array}{c} \varphi \end{array}$ on $\begin{array}{c} \pi_{mi} \end{array}$.

**Fig 21:**
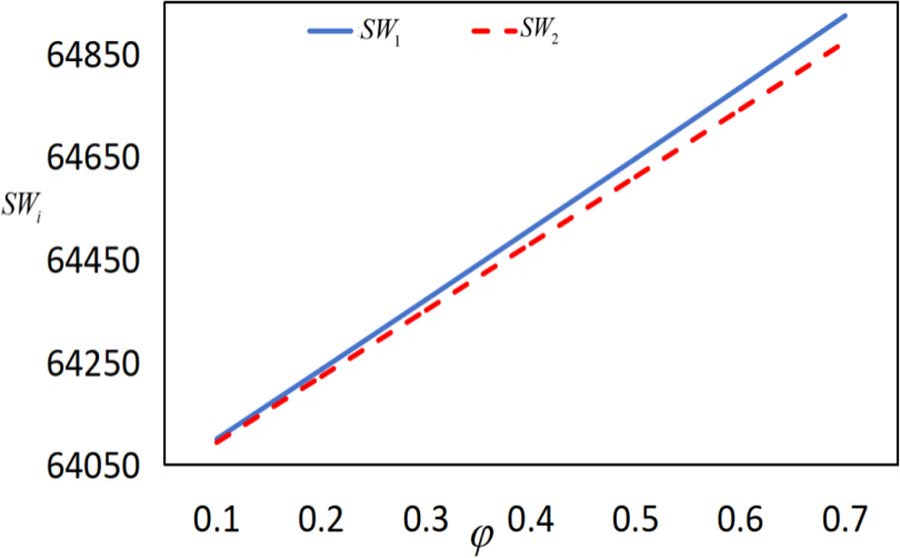
The impact of $\begin{array}{c} \varphi \end{array}$ on $\begin{array}{c} SW_{i} \end{array}$.

In conjunction with Table 2, it is straightforward to observe how the IoT application level, the economic benefits of the farmer and the company, as well as social welfare, vary with the government subsidy coefficient φ under the two subsidy mechanisms, as illustrated in Figs 18–21.

As illustrated in Fig 18, the IoT application level of the farmer increases under both subsidy policies with the increase in the intensity or the amount of government subsidies. However, the IoT application level under the interest rate subsidy policy is lower than that under the cost subsidy policy. It can also be observed that as the interest rate subsidy changes, the difference in the IoT application level between the two different subsidy mechanisms becomes increasingly larger, and the change in the IoT application level under the interest rate subsidy scenario is relatively small. In other words, when the government expenditure is equal, the cost subsidy policy is more favorable for the popularization of IoT.

As illustrated in Figs 19 and 20, the profits of both the farmer and the company increase under both subsidy policies as the intensity of government subsidies increases. However, the profits of the farmer and the company under the cost subsidy policy are higher than those under the interest rate subsidy policy. When the government expenditure is equal, the farmer is more inclined to accept the cost subsidy policy.

As shown in Fig 21, social welfare increases as the government continuously enhances the intensity of subsidies. Under the same level of government expenditure, social welfare is higher under the interest rate subsidy policy. Figs 19–21 reveal that as the subsidy coefficients increase, the differences in IoT application levels, the profits of the farmer and the company, and social welfare between the two different subsidy mechanisms become increasingly larger. This indicates that increasing the intensity of subsidies also magnifies the differences in the effectiveness of the two subsidy mechanisms. In summary, if the goal is to increase social welfare, the government should adopt the interest rate subsidy policy; if the goal is to promote the popularization of IoT and assist impoverished farmers, the government should adopt the cost subsidy policy.

## 6. Research implications

Firstly, farmers need to balance the positive effects of IoT investment with financing and acquisition costs to maximize benefits. Farmers should proficiently utilize IoT technology to increase crop yields and reduce costs associated with agricultural materials and labor. It is crucial for farmers to invest in IoT technology judiciously, as excessive acquisition costs can exacerbate their financial burden. When faced with two alternative subsidies from the government, if the financing interest rate is excessively high, farmers should opt for the interest rate subsidy and reduce IoT investment and production to safeguard their profits.

Secondly, when the financing interest rate is high, companies may need to lower the purchasing price of agricultural products to mitigate losses due to insufficient production. When government subsidies are available, companies can motivate farmers to adopt IoT technology by increasing the purchasing price of agricultural products. Furthermore, when faced with two alternative subsidies, if the financing interest rate is high, companies should set a purchase price higher than what would be offered under a cost subsidy policy to encourage farmers to choose the interest rate subsidy.

Thirdly, while government subsidies positively affect the application level and performance of IoT in the contract-farming supply chain, government resources are finite, and subsidy policies are goal-oriented. When devising subsidy policies, the government must consider five aspects: farmers’ benefits, companies’ benefits, banks’ benefits, government expenditure, and environmental benefits. If the goal is to increase social welfare, the government should adopt the interest rate subsidy policy; if the goal is to promote the popularization of IoT and assist impoverished farmers, the government should adopt the cost subsidy policy.

## 7. Conclusions

This paper integrates Internet of Things (IoT) technology into the contract-farming supply chain with financing considerations, constructs decision models across three subsidy scenarios—no subsidy, interest rate subsidy, and cost subsidy—and examines the application level of IoT technology and purchasing prices under these scenarios. It further explores the impact of bank interest rates, cost-effectiveness factors, and subsidy rates on the decisions and performance of various entities within the supply chain. The analysis also evaluates the effects and options of government subsidies with the same expected goals, yielding the following research conclusions:

Firstly, an escalation in bank interest rates negatively impacts the level of IoT application, purchasing prices, and the benefits of both farmers and companies. The higher the bank interest rate, the greater the financing costs incurred by farmers. Moreover, an increased financing interest rate diminishes the farmer’s inclination to invest in IoT, thereby affecting the extent of IoT adoption and agricultural production. When the financing interest rate surpasses a certain threshold, the benefits for both farmers and companies under the interest rate subsidy policy exceed those under the cost subsidy policy.

Secondly, under the scenarios of no subsidy, interest rate subsidy, and cost subsidy, the application level of IoT and the buyer’s profit are positively correlated with both the yield effect factor and the cost effect factor of IoT technology. When the cost-reducing effect of IoT is small, the use of IoT can actually increase the burden on the farmer. In other words, the benefits generated by IoT are not sufficient to offset the costs incurred. However, when the cost-reducing effect of IoT is substantial, the use of IoT can bring positive returns to the farmer, who will then be more inclined to invest in IoT. Although Wang et al. [11] and Gao et al. [13] argue that IoT can not only reduce the use of fertilizers and water but also effectively decrease labor management costs, they do not discuss the degree of the cost-reducing impact of IoT. If the impact is minimal, the farmer is unlikely to invest.

Thirdly, government policies such as interest rate subsidies and cost subsidies for IoT technology can encourage farmers to adopt a higher level of IoT technology, thereby enhancing the benefits of farmers and companies. However, such subsidies indirectly increase purchasing prices. When the government’s subsidy expenditure is equal, if the goal is to increase social welfare, the government should adopt the interest rate subsidy policy; if the goal is to promote the popularization of IoT and help impoverished farmers, the government should adopt the cost subsidy policy.

## 8. Future research

This study only considered the decision-making behavior of risk-neutral farmers and companies under government subsidies. In reality, farmers often exhibit risk aversion, and future research could explore risk-averse decision-making behavior among farmers. In scenarios without greenhouses or controlled environments, the actual output of agricultural products often deviates from the expected output due to uncertainties such as weather, seasons, and pests. This deviation results in output stochasticity risk, where the actual output is a random variable *x*, representing the fluctuation in agricultural output. The range of *x* is $\left[a_{1},a_{2}\right]$, with a probability density function f(·) and a strictly increasing and differentiable cumulative distribution function F(·). The expected value and variance of *x* are EX=μ, DX=$\sigma^{2}$, respectively. When farmers exhibit risk-averse behavior, their utility function under the Conditional Value at Risk (CVaR) criterion is given by $CVaR_{\eta }(\pi_{f0})=\max\{\delta +\frac{1}{\eta }E[\min(\pi_{f0}-\delta ,0)]\}$. Here, *E* denotes the expectation of the random variable, δ represents the Value at Risk (VaR) at a confidence level η, which is the upper bound of possible losses; η∈[0,1] indicates the degree of risk aversion, with higher values of η implying lower sensitivity to risk, and vice versa. When η = 1, it signifies that the farmer is risk-neutral. Additionally, contracts such as cost-sharing and revenue-sharing could be considered to further coordinate the supply chain.
